# Using a picture (or a thousand words) for supporting spatial knowledge of a complex virtual environment

**DOI:** 10.1186/s41235-023-00503-z

**Published:** 2023-07-25

**Authors:** Allison J. Jaeger, Steven M. Weisberg, Alina Nazareth, Nora S. Newcombe

**Affiliations:** 1 Department of Psychology, Mississippi State University, P.O. Box 6161, Mississippi State, MS 39762 USA; 2 University of Florida, Gainesville, USA; 3 Temple University, Philadelphia, USA

**Keywords:** Virtual navigation, Spatial cognition, External representations, Sketch maps

## Abstract

External representations powerfully support and augment complex human behavior. When navigating, people often consult external representations to help them find the way to go, but do maps or verbal instructions improve spatial knowledge or support effective wayfinding? Here, we examine spatial knowledge with and without external representations in two studies where participants learn a complex virtual environment. In the first study, we asked participants to generate their own maps or verbal instructions, partway through learning. We found no evidence of improved spatial knowledge in a pointing task requiring participants to infer the direction between two targets, either on the same route or on different routes, and no differences between groups in accurately recreating a map of the target landmarks. However, as a methodological note, pointing was correlated with the accuracy of the maps that participants drew. In the second study, participants had access to an accurate map or set of verbal instructions that they could study while learning the layout of target landmarks. Again, we found no evidence of differentially improved spatial knowledge in the pointing task, although we did find that the map group could recreate a map of the target landmarks more accurately. However, overall improvement was high. There was evidence that the nature of improvement across all conditions was specific to initial navigation ability levels. Our findings add to a mixed literature on the role of external representations for navigation and suggest that more substantial intervention—more scaffolding, explicit training, enhanced visualization, perhaps with personalized sequencing—may be necessary to improve navigation ability.

**Supplementary Information:**

The online version contains supplementary material available at 10.1186/s41235-023-00503-z.

## Significance statement

Navigation is an important skill across many facets of life. However, people vary greatly in their ability to generate accurate internal cognitive maps. People often rely on external representations like maps, written directions, or navigation technologies to help them navigate, but it is unclear if these external representations can help people to develop better spatial knowledge or build more accurate cognitive maps. Some research has indicated that spatial thinking skills are malleable and can be improved through training, but most of this work has examined small-scale spatial skills as opposed to large-scale spatial skills like navigation. In the present set of studies, we examined the impact of various external representations for supporting spatial knowledge development using a large-scale virtual environment. Whether participants had to generate their own maps or verbal descriptions (Study 1) or were given maps or verbal directions (Study 2), we found limited evidence indicating differential improvement in spatial knowledge. These findings suggest that training large-scale spatial skills like navigation may be challenging. Interventions using external representations for navigation may require more scaffolding, explicit training, or enhanced visualization to improve navigation ability.

## Introduction

To effectively navigate throughout one’s environment, we often rely on internal representations of the spatial structure of the world (Peer et al., 2021). One of the most well-known hypotheses regarding how mobile organisms navigate suggests that they generate and rely on internal cognitive maps. According to this cognitive map view of navigation, space is represented in the form of a metric, allocentric cognitive map that codes spatial properties such as location, orientation, boundaries, and distance and can be used, like a physical map, for the flexible planning of routes (Cheeseman et al., 2014; Ishikawa, 2023; Wang et al., 2023). While this view is widely endorsed by many navigation researchers (neuropsychological and behavioral), the term cognitive map is not without controversy (Newcombe, 2023; Weisberg & Newcombe, 2018). A popular alternative view is that rather than a map-like form, spatial knowledge takes a graph-like form made up of nodes and links without a global reference frame (Chrastil & Warren, 2014; Ericson & Warren, 2020; Warren, 2019).

Further, some take an individual-differences approach to examining navigation and suggest that *both* map-like and graph-like knowledge structures may be encoded in the brain (Peer et al., 2021). From this view of navigation, people differ in the kinds of spatial representations they can form. A substantial body of research has demonstrated that people differ considerably in their ability to learn large-scale environments and navigate within them (Hegarty & Waller, 2005; Ishikawa, 2023; Ishikawa & Montello, 2006; Nazareth et al., 2019; Weisberg & Newcombe, 2016, 2018; Wolbers & Hegarty, 2010). Together, these studies have demonstrated that while some participants may encode accurate internal maps, many others may encode spatial information imperfectly, in fragments, or rely entirely on location memory and route-based strategies for navigation.

### How are internal cognitive maps measured?

One challenge to studying internal cognitive maps is having accurate ways to measure them. The long-dominant framework for understanding spatial knowledge has suggested that it can be organized into landmark knowledge, route knowledge and survey or configuration knowledge (Siegel & White, 1975). Landmark knowledge includes information about salient or target locations, but without reference to their spatial locations. For example, one may know there was a library in the environment they navigated through but have no knowledge of its location within that environment or in reference to other landmarks in the environment.

Route knowledge includes information about the sequence of actions required to get from one point to another. According to Werner et al. (1997), route knowledge may exist in two forms, a very simple form that includes a series of landmarks and the direct connections between them ignoring all surrounding information, versus a more elaborate form that includes contextual and surrounding information**.** For example, knowing the path one needs to take to get from the library to the school may reflect a more simplistic form of route knowledge. On the other hand, a more elaborate form of route knowledge might result by integrating schemata and prior knowledge into the mental representation. For example, one may know that a particular town has a primary single street, or main street, from which other roads lead off to the sides. This scheme can then be enriched by specific features during navigation such as noticing that there is a small park at the corner, or that a road runs parallel to the path followed. Importantly, Werner et al. (1997) suggest that route knowledge is often accessed sequentially, that the number of paths emanating from each location is small, and that an egocentric, or view-based, reference system is used to decide where to go from any location.

Survey knowledge is typically regarded as an integrated form of representation with route-independent access to individual landmarks or locations (Montello, 1998). In particular, survey knowledge includes information about the configural organization of locations in an environment and is organized in a global, often allocentric coordinate system. This type of representation enables the inference of spatial relationships between arbitrary pairs of locations and can allow faster access to the locations of individual landmarks because the knowledge does not need to be accessed sequentially. For example, one may know that the library is west of the school and the grocery store is east of the school, and that both the library and the grocery store are further north than the school. Because of this integrated configural knowledge, one can point in the direction of, or take a novel shortcut to, one landmark from another even if they have never done so before.

To measure route knowledge, some have used route retracing tasks such as having people navigate unassisted through a route a second time or trace the individual routes they took during navigation on a map (e.g., Ishikawa et al., 2008; Meneghetti & Pazzaglia, 2021; Meneghetti et al., 2016; Parush & Berman, 2004). Other measures of route knowledge include making distance or directionality judgments for landmarks that were present within a single route (Gärling et al., 1981; Herman et al., 1987; Klatzky et al., 1990). These kinds of tasks are thought to tap into route knowledge because they do not require knowledge about the greater global configural organization of the environment but do require more than simple recollection of the landmarks that were present. And in fact, researchers (e.g., Lynch, 1960; Rand, 1969) have observed that people can be familiar with two separate regions but may not understand the spatial relationship between them.

To measure survey knowledge, or configural knowledge, the measures often require participants to integrate the spatial information of multiple routes into a single representation. Tasks of this sort include generating sketch maps of the entire environment, completing configural maps by placing target landmarks in their relative locations, orientation pointing tasks that require direction estimation between landmarks, and shortest path or shortcut tasks that asks participants to find novel pathways between landmarks (Bennett, 1996; Ishikawa et al., 2008; Meneghetti & Pazzaglia, 2021; Meneghetti et al., 2016; Parush & Berman, 2004; Weisberg & Newcombe, 2018; Weisberg et al., 2014). These kinds of tasks are thought to tap into survey knowledge because they require integrated knowledge about multiple routes and the relative directions between different locations even when there is no direct route between them—a task that can be difficult (if not impossible) to solve without relying on a flexible and allocentric spatial map.

It is, however, important to note that alternative conceptions of the structure and developmental course of spatial knowledge have been proposed. For example, Montello (1998) proposed a framework for understanding spatial knowledge that differed in a few important ways from the dominant framework proposed by Siegel and White (1975). While Montello does not disagree that there may be three types of spatial knowledge, he suggests that all three types of knowledge may be acquired simultaneously, rather than sequentially. Siegel and White suggest that spatial knowledge changes qualitatively as familiarity and exposure increase; first people acquire landmark knowledge, then route knowledge, and then finally survey knowledge. Montello, on the other hand, suggests that spatial knowledge increases quantitatively;  one can acquire all types of knowledge at once, with each type increasing relatively continuously as familiarity and exposure increase. In support of Montello’s framework, research has shown that even with only minimal exposure to a new environment, people can perform tasks that require some metric configurational knowledge using tasks such as taking shortcuts, returning directly back to starting locations, and estimating distances and directions directly between places (e.g., Gärling et al., 1981; Herman et al., 1987; Klatzky et al., 1990). All of this is simply to say that despite decades of research on cognitive mapping and spatial knowledge, it remains a challenge to clearly differentiate between different types of spatial knowledge and that there may be no stage at which only pure landmark or route knowledge exists, and where no metric information about distance and direction is included.

### External representations for supporting navigation

Whether in real or virtual space, having an accurate cognitive map is fundamental for being able to quickly and efficiently determine where something is and how one would get there. Because there is considerable variation in people’s ability to navigate (Hegarty & Waller, 2005; Ishikawa & Montello, 2006; Weisberg & Newcombe, 2018; Wolbers & Hegarty, 2010), with navigation a challenging task for many individuals, it is important to understand the utility of various kinds of external representations for various users in various situations. External representations are powerful tools for supporting and augmenting complex human behavior. Writing is the most common example, but visually based external representations such as diagrams, graphs, and maps also provide many advantages for learning and memory. They constrain learner’s attention to more relevant information (Stenning & Oberlander, 1995), make use of perceptual processes by grouping information and increasing the salience of important features (Tversky et al., 2006), and reduce memory demands by externalizing information needed for problem solving (Larkin & Simon, 1987).

Both verbal and visual communication is used to support the important daily task of navigation (e.g., maps, verbal directions, and linguistic or iconic signs). Different formats convey different types of information—a map is appropriate for calculating the distance and direction between places, while a set of verbal directions gives step-by-step instructions. Cognitive maps can be acquired through verbal or text-based descriptions of routes or environments and the spatial mental models developed from text may resemble the cognitive maps developed during navigation (Brunye et al., 2007; Giudice et al., 2007; Picucci et al., 2013; Taylor & Tversky, 1992). Importantly, research has indicated that people generally navigate more efficiently and effectively when given external supports like maps and verbal directions than when navigating without these tools (Hund & Minarik, 2006; Ishikawa et al., 2008; Krukar et al., 2020; Lowen et al., 2019; Munzer et al., 2020; Saucier et al., 2002). Yet, despite their usefulness in aiding navigation behavior while they are available, such external supports may be less useful in helping people build spatial knowledge. Even well-designed maps and verbal directions may not support survey or configural knowledge development if navigators do not integrate the provided external supports with their own representations of the environment. And in some cases, even if people do try to integrate the representations, they may fail to build the appropriate connections, resulting in an inaccurate or incomplete internal cognitive map.

An alternative option to simply being given a map or set of verbal directions to aid in configural knowledge development is to generate one’s own map or set of verbal directions. A substantial body of research from the cognitive literature on text comprehension has demonstrated improved comprehension or learning when individuals generate their own visual representations (e.g., Fiorella & Zhang, 2018; Hellenbrand et al., 2019; Schmeck et al., 2014; Schwamborn et al., 2010; Van Meter & Firetto, 2013) or when individuals generate verbal representations like explanations (e.g., Bisra et al., 2018; Chi et al., 1994; Pressley et al., 1992; Renkl et al., 1998). The distinction between generation and reference is critical for the learning process. On the one hand, generating external representations may facilitate thinking through a problem. For example, research has shown that having students actively construct their own visual representations can support expository science texts comprehension (Fiorella & Zhang, 2018), mathematics problem-solving (Rellensmann et al., 2017), model-based reasoning in chemistry (Cooper et al., 2017), and penetrative thinking in geology (Jaeger et al., 2020). When learners construct representations on their own, they are actively involved in externalizing their mental representation, which includes the processes of selecting, organizing, and integrating the information in the given situation (Van Meter & Garner, 2005). Together, the processes engaged during this generative activity facilitate inferencing and provide students with diagnostic cues that can support more accurate self-assessment (Van Meter & Firetto, 2013).

In the context of configural knowledge development, people experience an egocentric perspective during the process of navigation (their view of walking through), but at test they are often expected to take an allocentric perspective (drawing an overhead map, knowing the direction of one building relative to another). Thus, the act of generating an external representation after navigation, especially an overhead, allocentric map-based representation that requires the learner to integrate their egocentric perspective with an allocentric perspective could support spatial integration and result in better performance on measures of route and survey knowledge. Further, during highly structured navigation tasks where navigators follow set routes that do not require goal-directed or active navigation, learners may not spontaneously integrate the spatial information from multiple routes into a single configuration. The act of generating an external representation may foster this integration process, especially for learners who are less likely to take on this process on their own.

On the other hand, generated external representations may not be accurate. A sketch map of an area could omit details, regularize spatial relations, or otherwise be incomplete or outright wrong. It has been suggested that generated external representations will only be effective if they are of high quality and accurately depict the important structural relations and/or processes (Fiorella & Zhang, 2018). For example, several studies have found support for the *prognostic drawing effect*—that is, the suggestion that there is a strong relationship between drawing quality and learning (Schwamborne et al., 2010). Referring to external representations does not typically have the issue that they are inaccurate; but any benefits to learning that occur through the creation of the external representation itself can also not be realized.

## Study 1

The aim of Study 1 was to examine the impact of generating an external representation on the acquisition of spatial route knowledge and survey knowledge of a virtual environment (VE) after an initial experience of navigating through it. Research has demonstrated improved comprehension or learning when individuals generate visual or verbal representations (Bisra et al., 2018; Chi et al., 1994; Fiorella & Zhang, 2018; Hellenbrand et al., 2019; Pressley et al., 1992; Renkl et al., 1998; Schmeck et al., 2014; Schwamborn et al., 2010; Van Meter & Firetto, 2013). Prior work on both sketch and verbal description generation has demonstrated that these kinds of activities can support mental model development and inference generation in text-based learning scenarios (Fiorella & Mayer, 2016), but their impact on learning a complex spatial environment has not yet been explored.

A second goal was to assess whether self-generated sketch maps and verbal descriptions measure participants’ spatial knowledge of a large-scale VE. Prior navigation research has used sketch maps as measures of spatial survey or configuration knowledge (e.g., Blades, 1990; Ishikawa et al., 2008; Krukar et al., 2020; Schinazi et al., 2013; Zhong & Kozhevnikov, 2016), but an open question is whether verbal descriptions can also serve as measures of spatial knowledge. Verbal descriptions are necessarily categorical rather than continuous (direction words and prepositions do not typically specify metric information) and present information sequentially rather than simultaneously. Unlike sketch maps, which portray spatial information continuously and simultaneously, verbal directions do not align well with the demands of representing a large-scale environment.

Overall, we had several predictions for this study. We predicted that participants who generated sketch maps of the VE would outperform those who generated verbal descriptions of the VE on two measures of spatial knowledge acquisition, (1) a between-route orientation pointing task and (2) a model-building task requiring recall of the relative spatial locations of the target buildings. We also predicted that the quality of information presented in the sketches/verbal descriptions would be significantly correlated with navigation performance, but that sketches would contain significantly more spatial configuration information about the VE than verbal descriptions. This prediction was based on the idea that drawing an allocentric map may foster a survey perspective, while generating a set of verbal directions may foster a route perspective.

Thus, using a VE called Virtual Silcton (Weisberg et al., 2014) with a between-participants design, we offered participants the opportunity to generate their own external representations after an initial navigation experience with the instruction that they should intend on communicating the spatial layout of the environment and the locations of the buildings within it to someone else. We varied whether the external representation participants generated was a set of verbal directions or a sketch map and designed an active control condition in which participants completed a word search with the building names. After generating the external representation, participants were given the opportunity to freely explore the VE again with the purpose being to update their internal cognitive maps and revisit places they struggled to represent in their maps or verbal directions prior to completing the spatial knowledge measures.

## Method

### Participants

A sample of 172 participants (*M*_age_ = 20.38 years, *SD* = 2.12 years; 59 male) enrolled in an undergraduate psychology course were recruited for the current study. Participants signed up for the study via SONA Systems, a university-wide online recruitment website and received course credit for participation. Participants were randomly assigned to one of three experimental conditions 1. An a priori power analysis using G*Power indicated that to conduct a one-way between subjects Analysis of Variance (ANOVA), we needed at least 159 total participants (or ~ 53 participants per group) to achieve power of 0.80, assuming a medium effect size of Cohen’s *f* = 0.25, and alpha of 0.05. We exceeded this minimum required sample size to accommodate all participants who had signed up via SONA.

## Materials

The experiment was administered on a Windows 7 64-bit computer with an Intel Core i5-6600 CPU @ 3.30 GHz and Nvidia GeForce GT 610 video card. The experiment was displayed on a 40 cm × 62 cm LCD monitor with a refresh rate of 60 Hz and resolution of 1680 X 1050.

### Virtual environment navigation tasks (Virtual Silcton)

**Fig. 1:**
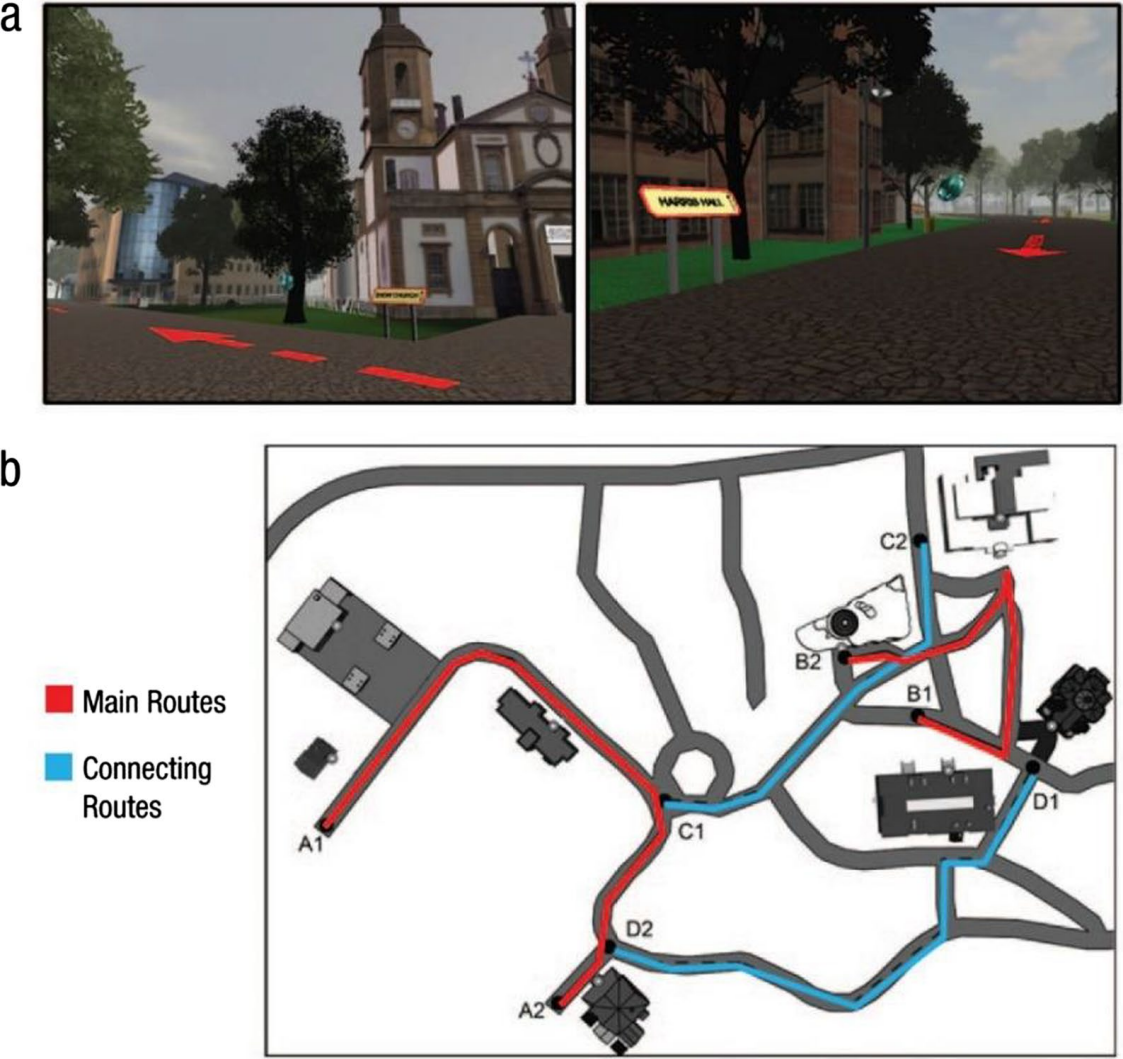
Examples from the Virtual Silcton desktop virtual environment used in Studies 1 and 2. *Note.* The top panel **a** shows screenshots from the Virtual Silcton desktop virtual environment; the bottom panel **b** shows an overhead map of the main routes and connecting routes participants navigated

Virtual Silcton (Fig. 1), a VE modeled on a real-world college campus built with Google Sketchup and Unity 3D, is an objective measure of navigation ability (Schinazi et al., 2013; Weisberg et al., 2014). Virtual Silcton was designed to replicate the saliency and spatial location of buildings and non-building objects like trees and trash cans, without replicating the exact architecture of the real-world structures (Schinazi et al., 2013).

In most prior studies using Virtual Silcton (e.g., Schinazi et al., 2013; Weisberg et al., 2014), learning involves virtually traveling along four routes: two main navigation routes that tour different areas of the environment and do not intersect each other (Routes A and B), and two connecting routes, which intersect both main routes (Routes C and D). In the present study, all participants navigated the two main routes and one connecting route (only Route C). The main routes were presented in random order across participants and the connecting route was always presented last. Along each main route participants learn the names and locations of four unique target buildings (eight total). Connecting routes do not contain new target buildings but instead offer an opportunity for participants to learn how the two main routes are connected. Participants travel along routes using the mouse (to look) and arrow keys (to move) to travel along route paths, which are indicated by red arrows and from which participants are restrained from leaving by invisible walls. Target buildings are indicated by the presence of a blue gem hovering along the route near the building and named with a yellow and red sign. Participants traveled from the start of each route to the end and then back to the start. After learning the four target buildings on each main route, participants learned how the eight target buildings were connected by walking along the connecting route.

After learning, participants completed two measures—a pointing task and a model-building task (Weisberg et al., 2014). These measures assessed the participant’s ability to create accurate and integrated representations of the VE.

#### Pointing task

In the pointing task, participants were positioned next to one of the eight target buildings and were prompted to point in the direction of each of the other seven buildings. Participants pointed by rotating a virtual crosshair on the horizontal plane using the mouse in the direction of the front door of the target building and recorded their response by clicking. They were instructed to specifically point their crosshair at the front door of each building, and to be careful to only click once to record their answer. This process was repeated for each of the eight buildings for a total of 56 pointing trials. A pointing error score for each participant was calculated based on the absolute value of the participant’s answer minus the correct answer. If that value exceeded 180, we corrected it by subtracting the value from 360.

Performance on the pointing task was subdivided into within-route and between-route pointing performance based on the relation between the target building and the participant’s pointing location (i.e., whether the current location and target building initially occurred on the same main route or on different main routes). The within-route pointing trials measure the participant’s ability to locate one target building relative to another target building where a direct route was present during navigation. Thus, the within-route error score was calculated for trials where the target building was on the same route as that of the participant. Because these trials do require distance and direction estimation, but do not require integration across multiple routes, it could be conceptualized as a measure of elaborate route knowledge or more local (rather than global) configural knowledge. The between-route pointing trials were meant to assess more global survey or configural knowledge because they required integrated knowledge about multiple routes and directionality judgments between locations where no direct route between them was experienced. The between-route error score was calculated for trials where the target building was on a different main route as that of the participant. There were 24 within-route trials and 32 between-route trials. Due to a bug affecting pointing data collection at the time of this study, the pointing data were corrected in the manner described in Weisberg and colleagues (2022).

#### Model-building task

In the model-building task, participants were told that they would construct a map of the VE using a bird’s eye view. Aerial-view images of the eight target buildings were arrayed at the bottom of a blank black rectangle in a web browser. To create the map, participants could move each building to the position in the rectangle they believed it to be located relative to the other buildings. Participants were instructed that the orientations of the images and the orientation of the map would not be part of their score. Hovering over each image of the building displayed a front-view image of the building on a single-color background and the name of the building. We scored the maps using bidimensional regression analysis (Friedman & Kohler, 2003; Tobler, 1994) which resulted in an *R*^2^ for each participant. The *R*^2^ value can be interpreted as the percent of variance explained in the actual configuration of buildings by the participant’s map, accounting for differences in scale, translation, and rotation in the overall configuration. The model-building task served as an additional measure of global survey or configural knowledge because it required an integrated understanding of the relative locations of all landmarks within the environment.

#### Virtual Silcton procedure

Participants navigated through Virtual Silcton, but in the present study, participants experienced four phases: learning; externalization generation; free exploration; and testing.

In the learning phase, participants were first instructed to learn the names and locations of each of the eight target buildings by walking along the two main routes in the VE. Route paths were indicated by red arrows, and target buildings were indicated by the presence of a blue gem hovering near each target building. Participants walked from the start of each route to the end and then back to the start. After learning the four target buildings on each main route, participants learned how the eight target buildings were connected by walking along one connecting route (connecting route C for all participants).

Immediately after completing the connecting route, participants were randomly assigned to an externalization condition: sketch map, verbal description, or control. In the sketch map condition, participants were given a piece of paper with a pre-drawn rectangle outline with the following instructions, “In the box below, please sketch a map of the virtual environment you just explored so that another person could use your sketch to find the 8 target buildings. Please label the buildings with their names if you can.” In the verbal condition, participants were given a piece of paper with the same pre-drawn outline but were instructed to, “Write a summary in the box describing the virtual environment so that another person could use your description to find the 8 target buildings.” In the control condition, participants were given a word-search puzzle to solve. The word search included the names of the 8 target buildings, but these names were not provided for the participant. Rather, participants were instructed to, “Try and remember the target building names from the VE and find and circle the names in the word search puzzle.” In all three conditions, participants were given 4 min to complete the activity.

After completing the externalization task, participants went into the free exploration phase. The participant was placed in a starting location toward the center of Virtual Silcton without red arrows on the routes or invisible walls. Participants were instructed that the purpose of the free exploration phase was not to learn any new buildings, but rather was to, “… move anywhere in the environment to try and fill in gaps in your knowledge about the relations between the 8 target buildings.” Prior research on learner-generated drawing has indicated that it is most supportive for learning when students are able to receive feedback on their drawings or are given the opportunity to compare their drawings to an ideal drawing (Gagnier et al., 2017; Schwamborne et al., 2011; Van Meter et al., 2006; Zhang & Fiorella, 2019). Thus, the goal of the free exploration phase was to give students the opportunity to freely navigate through the environment and revisit any specific areas they struggled to represent in their externalization activities 2. Participants were given a maximum of 5 min to freely explore, after which they moved to the testing phase. In the testing phase, participants completed the within- and between-route pointing tasks and the model-building task as measures of spatial knowledge acquisition.

#### Sketch map and verbal description coding

The sketch maps and verbal descriptions were coded for quality and inclusion of key elements. Sketch maps and verbal descriptions were assessed on two parameters: (1) *target details* represented the number of target buildings sketched or described and served as an indicator of landmark knowledge and (2) *route details* represented the extent to which a participant attempted to integrate the two routes into a unified representation in their externalization. The score for *route details* was modeled after the sketch quality scoring developed by Krukar et al. (2020). In their scoring, categories ranged from 0 to 3, where 0 represented sketch maps that were nonsense, or not maps, and 3 represented sketch maps that were good and might allow a navigator to use the map in practice. Because we wanted to develop a scoring system that could be used for both sketch maps and verbal descriptions, adaptations were required. For our purposes, a score of 0 was given if the externalization included no route information and only listed building names (thus could not be used for navigation at all), a score of 1 was given if only a single route was represented or described, a score of 2 was given if two routes were represented or described but no attempt to integrate the routes was made, and a score of 3 was given if the two main routes were represented or described and the participant attempted to integrate the two routes into a single representation by depicting or describing the connecting route. This scoring did not consider the spatial accuracy or correctness of participants externalizations, but rather was meant to capture their attempts at integrating all the information into a single mental representation. Thus, it was possible for a participant to receive the highest score for *route details* without including all 8 target buildings in their externalization. While it was possible to code for target details in the control condition (number of building names found in the word search), it was not possible to code for route details in this condition, and thus, only the sketch map and verbal description conditions have scores for this variable. As an initial step before coding, two research assistants followed a detailed guideline to independently code these two variables for 30 participants (10 participants per group). This independent coding resulted in a high inter-rater reliability (percent agreement = 90% for sketch maps and verbal descriptions), and the disagreements were resolved by the research team prior to completing the coding for all externalization activities.

#### Sketch accuracy

In addition to analyzing the amount of target and route details contained in participants’ externalizations via the hand scoring methods previously described, sketch map accuracy was also assessed using the Gardony Map Drawing Analyzer (GMDA; Gardony et al., 2016), a software tool for sketch map analysis. The GMDA allowed us to score all the drawings along an additional parameter—their spatial accuracy. Specifically, the GMDA uses a graphical user interface to measure the configural accuracy of the sketch map compared to a complete and accurate provided map using bidimensional regression parameters (Friedman & Kohler, 2003) and novel parameters measuring configurational accuracy while simultaneously accounting for the number of landmarks portrayed by the participant (Canonical Organization). The GMDA provided two measures of sketch map accuracy: (1) the number of landmarks missing in the sketch maps compared to the correct provided map and (2) the spatial accuracy of the routes and landmarks compared to the correct provided map. Thus, the GMDA score also assessed both landmark knowledge and global survey or configural knowledge.

## Psychometric and self-report measures

All participants completed a battery of six psychometric and self-report measures. These included one measure of verbal ability, two measures of working memory capacity, one measure of spatial orientation skill, and two spatial thinking self-report measures. 

### Wide Range Achievement Test, Word Reading Subtest (WRAT-4)

The WRAT-4 Word Reading Subtest (Wilkinson & Robertson, 2006) is a measure of verbal IQ that correlates very highly with the WAIS-III and WISC-IV. This test has been norm-referenced with reliability coefficients ranging from 0.87 to 0.93. This test requires participants to pronounce 55 individual words. Scores represented the number of words pronounced correctly out of 55.

### Running span

The running span is a working memory capacity (WMC) measure based on a version used previously by Broadway and Engle (2010). Broadway and Engle showed this measure to be highly reliable and correlated with other measures of working memory capacity. In this task, participants are asked to remember the last few letters of a rapidly presented series. For each trial, participants see between 4 and 9 letters presented via computer, and must remember the last 3 to 6 letters, with three trials of each length. Letters are presented for 300 ms, with a 300 ms blank screen between each letter. Scores were computed as the number of letters recalled in the correct serial position.

### Backwards digit span

The backwards digit span involves remembering a series of numbers in reverse order and was adapted from previous work examining WMC (Oberauer et al., 2000; Cronbach’s *α* = 0.81). Participants see a string of numbers between 2 and 8 digits in length presented individually via computer and are asked to type back the numbers in reverse order leaving a blank for any number they cannot remember. Numbers are presented for 500 ms, with a 500 ms blank screen between each number. Numbers are presented in sets of increasing size, with two trials of each size. Scores were computed as the total number of digits recalled in the correct serial position.

### Spatial Orientation Test (SOT)

The SOT (Hegarty & Waller, 2004; Cronbach’s *α* = 0.83) is a revised version of the test used by Kozhevnikov and Hegarty (2001) and tests the ability of participants to imagine different perspectives and orientations in space. Participants saw an array of two-dimensional objects drawn on a sheet of paper and were asked to imagine that they were standing at one object with a specific facing orientation. Their task was to draw an arrow from this spatial location and orientation to a third object in the array. There were 12 items, and participants were given 5 min to complete the test. Participants’ score on the test (SOT error) was computed as the average of the absolute difference in angle between the correct response and the participant’s response. If that value exceeded 180, we corrected it by subtracting the value from 360.

### Object-Spatial Imagery and Verbal Questionnaire (OSIVQ)

The OSIVQ is a self-report questionnaire designed to assess individual differences in cognitive styles (Blazhenkova & Kozhevnikov, 2008). The questionnaire is comprised of 45 items that are designed to distinguish between three different cognitive styles: (1) object imagers (e.g., “My images are very colorful and bright”), (2) spatial imagers (e.g., “I was very good in 3D geometry as a student”), and (3) verbalizers (e.g., “My verbal abilities would make a career in language arts relatively easy for me”). Participants rated each item on a 5-point Likert scale (1—strongly disagree; 5—strongly agree). For each participant, the 15 item ratings from each factor were averaged to create an object score (Cronbach’s *α* = 0.83), a spatial score (*α* = 0.79), and a verbal score (*α* = 0.74), where higher scores indicate greater agreement.

### Santa Barbara Sense of Direction Scale (SBSOD)

The SBSOD is a self-report measure that assesses an individual’s ability to orient oneself in an environment (Hegarty et al., 2002). The questionnaire is comprised of 15 items (e.g., "I very easily get lost in a new city") that require responses on a 7-point Likert scale (Cronbach’s *α* = 0.90). This task was scored by reverse scoring the positively phrased items, summing the scores for all the items together, and then dividing the total by the number of items. This scoring method results in scores ranging between 1 and 7 where higher scores indicate a better perceived sense of direction.

## Procedure

Participants were run individually and were randomly assigned to one of three experimental conditions—sketch map, verbal description, and control. Participants in all three conditions first completed the WRAT and a short demographic survey. Next, participants were introduced to the VE and practiced navigating in the environment using the mouse and arrow keys. All participants completed the first two main routes, which were counterbalanced across participants, and one connecting route, which was the same for all participants. After navigating all three routes in the VE, participants were given 4 min to complete the externalization activity (sketch, verbal description, or word search control). Next, participants went back into the VE to complete the free exploration phase, followed by the testing phase in which they completed the pointing and model-building tasks. Lastly, participants completed the battery of psychometric and self-report measures. All participants completed these measures in the same order and format starting with the SOT, then the OSIVQ, then the SBSOD, and finally the working memory measures. The two working memory measures were combined into a composite working memory capacity (WMC) score. Upon completion of these measures, participants were debriefed and thanked for their participation. The study sessions were completed in approximately 1 h.

## Results

All frequentist analyses were conducted using IBM SPSS Statistics Version 28. In addition to these analyses, Bayes’ factors were also computed using the BayesFactor package in R (Morey, 2015). Bayes’ factors quantify the support for one hypothesis over another, e.g., support for the alternative hypothesis compared to support for the null hypothesis. Bayes’ factors can be interpreted as the ratio of evidence for the alternative hypothesis to evidence for the null hypothesis, meaning values > 1 indicate support for the alternative hypothesis and values < 1 indicate support for the null hypothesis (Masson, 2011; Rouder, 2012).

To ensure there were no baseline differences across the three experimental conditions, we conducted one-way ANOVA tests with condition as the independent variable and our psychometric and self-report measures as dependent variables. Because scores on the running span and backwards digit span tasks correlated significantly with each other, *r*(164) = 0.57, *p* < 0.001, participants’ scores were converted to z scores and averaged together to form a composite WMC measure. Analyses revealed no baseline differences in WMC (*F*(2, 157) = 0.08, *p* = 0.93, *ƞ*^2^ = 0.001, BF_10_ = 0.007), verbal fluency (*F*(2, 162) = 0.35, *p* = 0.71, *ƞ*^2^ = 0.004, BF_10_ = 0.008), sense of direction (*F*(2, 164) = 2.00, *p* = 0.14, *ƞ*^2^ = 0.024, BF_10_ = 0.04), spatial orientation (*F*(2, 164) = 2.45, *p* = 0.09, *ƞ*^2^ = 0.029, BF_10_ = 0.065), or cognitive style (object: *F*(2, 131) = 1.45, *p* = 0.24, *ƞ*^2^ = 0.022, BF_10_ = 0.03; spatial: *F*(2, 131) = 0.17, *p* = 0.85, ƞ^2^ = 0.003, BF_10_ = 0.009; verbal: *F*(2, 131) = 0.53, *p* = 0.59, *ƞ*^2^ = 0.008, BF_10_ = 0.012) as a function of externalization activity condition. See Additional file 1: for descriptive statistics for all psychometric and self-report measures (Additional file 1: Table S2).

### Externalizing knowledge did not improve navigation performance

To assess whether generating externalizations of one’s internal cognitive maps would impact spatial knowledge acquisition, a series of one-way between subject ANOVAs were conducted. Specifically, we investigated the effect of externalization activity condition (sketch map, verbal description, control) on within-route pointing error, between-route pointing error, and model building 3. There were no significant differences between the three experimental groups in within-route pointing error, *F*(2, 165) = 1.88, *p* = 0.155, *ƞ*^2^ = 0.02, between-route pointing error, *F*(2, 165) = 0.39, *p* = 0.68, *ƞ*^2^ = 0.005, or model-building performance, *F*(2, 165) = 1.07, *p* = 0.34, *ƞ*^2^ = 0.013. These analyses revealed that externalizing alone did not significantly improve navigation performance in the VE (see Table 1). Despite non-significant omnibus ANOVAs, all three were followed up with pairwise contrasts to directly test the first hypothesis that participants in the sketch map group would outperform those in the verbal description group. There were no differences between any of the three groups on either the between-route pointing task or the model-building task (all *t*s < 1.45, *p*s > 0.14, all BF_10_ < 0.53). There were also no significant differences between groups on the within-route pointing task (*t*s < 1.93, *p*s > 0.06, all BF_10_ < 1.03).

**Table 1:** Descriptive statistics for navigation tasks by condition in study 1

	Sketch		Verbal		Control	
	*M*	*SD*	*M*	*SD*	*M*	*SD*
Within-route (error)	24.73	11.32	27.62	14.39	23.22	12.39
Between-route (error)	40.82	15.02	43.07	14.77	42.44	14.26
Model building	0.5475	0.2773	0.4694	0.2702	0.5010	0.2642

### Externalization activities did not demonstrate attempts at spatial integration

We analyzed the external representations for their inclusion of target buildings (*target details*) and how well they integrated information across routes (*route details*). Overall, sketch maps (*M* = 5.31, *SD* = 2.06) included significantly more target buildings from the VE than the verbal descriptions (*M* = 4.21, *SD* = 2.27), *t*(102) = 2.58, *p* = 0.01, *d* = .51, BF_10_ = 3.83. Further, when examining route information, verbal descriptions were less likely to include route information and (simply list target buildings), whereas sketches were more likely to depict information about the two main routes, *Χ*^*2*^(3, *N* = 104) = 27.14, *p* < 0.001, Cramer’s *V* = 0.51. This finding provides some support for the a priori prediction that sketches would contain more spatial configuration information about the VE than verbal descriptions. Few students in either condition integrated the routes or included the connecting route (see Table 2 for a frequency distribution of *route details* by condition).

**Table 2:** Group differences in the amount of externalized route details for Study 1

	Sketch	Verbal
0—List of target names and no routes	0	21
1—Representation of one route	29	15
2—Representation of two routes	18	11
3—Attempted integration of routes	5	5

### Externalized knowledge measured rather than improved spatial knowledge

Although there was no differential impact of externalization activity on navigation performance, we were interested in whether differences in the quality of externalized knowledge representations was related to navigation performance. To examine this, we conducted a series of bivariate correlations between Silcton performance measures with route and target details, separately for the sketch map and verbal conditions.

In the sketch map condition, the number of target details was significantly correlated with within-pointing (*r*(49) = − 0.35, *p* = 0.01, BF_10_ = 3.25) and between-pointing error (*r*(49) = − 0.29, *p* = 0.04, BF_10_ = 1.19), but not model building (*r*(49) = 0.20, *p* = 0.17, BF_10_ = 0.40). The route details score was significantly correlated with all Silcton performance measures (within-pointing error, *r*(49) = − 0.37, *p* < 0.01, BF_10_ = 4.79; between-pointing error, *r*(49) = − 0.52, *p* < 0.001, BF_10_ = 251.93; and model building, *r*(49) = 0.37, *p* < 0.01, BF_10_ = 4.79). In the verbal condition, the number of target details was significantly correlated with within-pointing (*r*(48) = − 0.36, *p* = 0.01, BF_10_ = 3.70) and between-pointing (*r*(48) = − 0.40, *p* < 0.01, BF_10_ = 8.39) error, but not model building (*r*(48) = 0.27, *p* = 0.06, BF_10_ = 0.87). The route details score was significantly correlated with within-pointing error (*r*(48) = − 0.30, *p* = 0.03, BF_10_ = 1.33), but not between-pointing error (*r*(48) = − 0.21, *p* = 0.14, BF_10_ = 0.43) or model building (*r*(48) = 0.19, *p* = 0.19, BF_10_ = 0.36). The number of target details and route details were correlated within condition as well. Participants who included more target details also included more route details for both the sketch (*r*(50) = 0.45,* p* = 0.001, BF_10_ = 34.14) and verbal description (*r*(50) = 0.58, *p* < 0.001, BF_10_ = 2896.73) conditions.

To further address if the quality of externalized knowledge representations would be significantly related to navigation performance, a linear regression analysis was conducted and indicated that the amount of target details contained in sketches and verbal descriptions significantly predicted unique variance in within-route pointing error and between-route pointing error but not model-building performance (see Table 3). This relation was not moderated by the type of externalization (i.e., there was no difference between good-quality sketches and good-quality verbal descriptions in explaining pointing error).

**Table 3:** Regression analysis for pointing and model-building performance in Study 1

	Within-route error			Between-route error			Model building		
	*B*	*SEM*	β	*B*	*SEM*	β	*B*	*SEM*	β
Sketch/verbal group	.16	3.70	.01	1.66	3.75	.04	.03	.06	.05
Target details	− 2.17	.98	− .26*	− 2.05	.99	− .24*	.02	.01	.14
Route details	− 4.09	2.49	− .19	-4.48	2.52	− .21	.06	.04	.19

*p < .05

### Quality of sketches reflects spatial knowledge

Did participants who produced more accurate sketch maps demonstrate better route or survey knowledge? We further assessed the configurational accuracy of the sketch maps using the GMDA, which provides several scores indicating the spatial accuracy of the map. The two measures considered in these analyses are Canonical Organization and Landmarks Missing. After optimal rotation, translation, and scaling of the sketch map to match the correct template, Canonical Organization measures configurational accuracy by comparing each landmark to every other and counting one point for each correct relative spatial position (e.g., if Landmark A is correctly placed above and to the right of Landmark B that pair receives 2 points). Comparisons to excluded landmarks count as zero points; thus, Canonical Organization penalizes sketches for missing landmarks (Gardony et al., 2016). Canonical Organization is the proportion (from 0 to 1) of correct spatial positions to all possible pairwise relations. Landmark Missing is merely the number of landmarks not included by the participant. Scores range from 0 (no buildings missing from hand-drawn map) to 8 (all 8 buildings missing from hand-drawn map).

First, we examined correlations between the hand-scored drawing quality variables and GMDA-generated quality variables. The correlation between *target details*, which represents the number target buildings depicted in the drawing, and *landmarks missing*, which represents the number of target landmarks excluded from the hand-drawn map, was significant (*r*(50) = − 0.84, *p* < 0.001, BF_10_ = 1.28E + 12) indicating high agreement between our hand scoring and the GMDA scoring. There was also a significant correlation between route details and canonical organization (*r*(50) = 0.44, *p* = 0.001, BF_10_ = 26.14).

We also examined correlations between drawing quality measures and VE performance measures (pointing and model building). As shown in Table 4, the hand-scored drawing quality measures show the same pattern of correlation with all VE performance measures as the GMDA quality measures. In general, participants who included more target buildings in their drawings demonstrated less error in their within and between-route pointing judgments. Target details and landmarks missing were not correlated with model-building performance. Also, both route details and canonical organization were significantly correlated with within- and between-route pointing error and model-building performance.

**Table 4:** Correlations between sketch quality measures and VE performance outcomes

	Within pointing	Between pointing	Model building
Target details (hand scored)	− 0.35* (3.24)	− 0.29* (1.19)	0.20 (0.40)
Landmarks missing (GMDA scored)	0.37** (4.79)	0.33* (2.26)	− 0.15 (0.26)
Route details (hand scored)	− 0.37** (4.79)	− 0.52** (251.93)	0.37** (4.79)
Canonical Org (GMDA scored)	− 0.30* (1.38)	− 0.40** (9.06)	0.29* (1.19)

For all reported correlations, n = 51. BF_10_ values for each correlation are indicated in parentheses. **p < .01, *p < .05

## Discussion

Our primary question for Study 1 was whether generating an external representation of a VE after an initial navigation experience would support spatial knowledge acquisition. We predicted that generating an allocentric map of the environment would support greater global survey or configural knowledge than generating a set of verbal instructions because sketch maps portray spatial information continuously and simultaneously whereas, verbal directions do not align well with the demands of representing a large-scale environment. Counter to this hypothesis, we did not observe better performance on within-route or between-route pointing tasks or model building for participants who generated sketch maps compared to participants who generated verbal descriptions or participants in the control condition.

Another primary question for this study was how the quality of information presented in the sketches/verbal descriptions would relate to spatial knowledge acquisition, and if sketches would contain more configural spatial information about the VE than verbal descriptions. The results from the present study provided partial support for these hypotheses. Sketches contained significantly more target buildings than verbal directions, indicating that participants in the sketch map group had greater landmark knowledge, which aligns with results obtained by Taylor and Tversky (1992). The sketches were also more likely to represent at least one route than the verbal directions, which were more likely to simply list building names without referencing the routes they were on or the spatial relations between the buildings. This result seems to indicate that sketching may have supported the development of greater route knowledge. However, we did not see that sketches included more configural or survey information than the verbal directions. This somewhat surprising result could have been due to none of the groups developing sufficient configural knowledge to put into their descriptions, or that they did not think to include that information, or that both included the same (high) level of configurational information. It is worth noting that although not as robust as the sketch maps, the verbal descriptions did have some spatial properties (Denis, 2018) and did appear to capture some of the participants spatial knowledge.

In terms of representation quality relating to the objective spatial knowledge measures, the number of target details (buildings) included in sketches and verbal descriptions was related to less within-route and between-route pointing error but was not related to model-building accuracy. One possible explanation for these findings might be that the existence of more landmarks in one’s internal mental representations leaves less room for error in the direction judgments. When looking at representation quality for only the sketch maps, both target details and route details were correlated with less between- and within-route pointing error and greater model-building accuracy. These results align with prior research demonstrating that sketch maps can reliably convey environmental knowledge and are valid predictors of navigational performance (Blades, 1990; Ishikawa et al., 2008; Krukar et al., 2020; Zhong & Kozhevnikov, 2016). Further, these results lend support to the *prognostic drawing effect* (Schwamborne et al., 2010) indicating that it was only the students who were able to generate high quality maps and verbal descriptions that demonstrated learning of the VE.

One interpretation for this effect is that generating external representations of one’s spatial knowledge is only useful if the external representation is accurate. This interpretation is consistent with the large individual differences in navigation behavior reported generally (Weisberg et al., 2014, 2018) and with experiments showing that verbal and visuospatial working memory may (Menghetti et al., 2021; Meneghetti & Pazzaglia, 2021; Wen et al., 2011) or may not (Blacker et al., 2017; Weisberg & Newcombe, 2016) play distinct roles in supporting distinct aspects of spatial navigation behavior. Individuals with low spatial thinking skills who have difficulty learning spatial environments to begin with may not be able to create accurate external representations from their inaccurate mental representations and will likely not be able to use their maps to improve. Thus, in Study 2, we examined the effect of providing accurate external representations of the VE during navigation by allowing participants to navigate using a set of schematic overhead maps or a set of route-based verbal directions.

## Study 2

The main goal of Study 2 was to examine the effect of providing accurate external representations in the form of schematic overhead maps or sets of route-based verbal directions of the VE during navigation. Several studies have compared the use of different types of external representations while navigating, but the types of verbal and visual media used in these studies and the types of recall or learning tasks administered have varied substantially. Thus, the relative benefits of verbal route representations versus visual map representations on spatial knowledge acquisition remain inconclusive.

Much of the research on verbal directions and maps has focused on supporting ongoing navigation, i.e., getting people where they want to go. In the case of using maps, they have been shown to have a positive impact on our ability to navigate both in real life navigation scenarios (e.g., Ishikawa et al., 2008; Münzer et al., 2012) and when navigating in VEs (e.g., Gardony et al., 2013; Münzer et al., 2020). Further, combining navigation-based and map-based information has been shown to support the development of good-quality mental representations (Siegel & White, 1975). Language can also be used to provide information about space (Denis et al., 1999), and some studies have compared various kinds of verbal instructions, e.g., the effectiveness of mentioning cardinal points (e.g., “to the north”) (Hund & Minarik, 2006; Saucier et al., 2002).

Several studies have directly compared the use of different types of external representations (maps or verbal instructions) while navigating. The results of these studies have been mixed, with some finding benefits for verbal route instructions, some finding benefits for maps and some finding benefits for both. In one of the first studies comparing the impact of verbal route instructions versus maps, Streeter et al. (1985) compared using a route map to audio recorded verbal instructions for guiding drivers in an unfamiliar environment. They found that using auditorily presented verbal instructions during navigation was better than using a route map in terms of travel time, distance, and the number of navigation errors. Similarly, Parush and Berman (2004) found that participants given a written verbal route list during an initial learning phase were able to navigate to target locations in a VE more quickly and more efficiently than participants given a map to use during the learning navigation phase. However, when these navigation aids were taken away during a transfer phase and participants had to navigate the environment without aid, those who initially used the map to support navigation were able to navigate more quickly and efficiently and made fewer errors in an orientation pointing task. Research by Pazzaglia and De Beni (2001) found that participants using a map during a direct-experience navigation task were able to navigate through a building more quickly and with less hesitation than those using written verbal route instructions. However, they found no differences in the number of errors participants made during navigation and importantly they did not include any transfer navigation tasks or orientation pointing tasks. Other work has found that both types of representations have a positive impact on recall of the environment learned. Schlender et al. (2000) found that navigation performance benefited from giving participants a map or verbal instructions during a virtual navigation task compared to having no external representation. Further, providing either of these representations during navigation was more beneficial than only presenting them prior to navigation.

Together, these results offer a mixed outlook on the impact of verbal and visual representations during navigation. Verbal instructions may be more beneficial than map representations while people are initially experiencing and forming landmark and/or route knowledge about an unfamiliar space, but maps may be more beneficial for developing survey knowledge or for supporting the integration of multiple types of spatial knowledge. In line with this idea, Meneghetti and Pazzaglia (2021) suggest that both types of representation can help navigators get to their destination, and that there may be advantages for language in some cases and visual representations in other cases. Auditorily presented verbal representations may be more beneficial in cases where there may be interference between watching the environment you are navigating through and watching a map. In these cases, they suggest that having to visually inspect a map during navigation would consume the visuospatial working memory resources needed for maintaining attention and focus on the environment.

There is less research on preparing for navigation by asking for directions or consulting a map, either ahead of a trip, or in a pause during navigation, perhaps when confused or lost. In this situation, the external representation provides a schema or framework into which sensory information from the trip can be fit, retrospectively, prospectively, or both. External representations might also improve the accuracy of the internal representations people form. In an initial study of this kind, Meneghetti and Pazzaglia (2021) found some advantages for viewing a map before navigation, compared to reading a verbal configural description, or no preparation. They found that participants who were given a map to study prior to navigation showed better performance on both a path drawing and pointing task than a navigation-only group. Further, the map group showed better performance on the path drawing task than a group that was given a verbal description before navigation. Interestingly, the groups all performed equally on a route retracing task that assessed route knowledge. The authors suggested these results indicate that seeing a map prior to navigation allows the learner to construct a mental representation that includes both allocentric information from the map and egocentric information from their navigation experience. However, their environment contained no landmarks or distinguishing features, and consisted of only 90° turns, thus the verbal description may have had fewer anchors on which to draw.

The goal of the present study was to further examine the differential effects of map representations and verbal descriptions on spatial knowledge acquisition. In Study 2, a group of participants completed an initial navigation experience where they all passively navigated through a VE with 2 main routes and 2 connecting routes. Approximately one week later, participants returned for a second navigation experience. During this experience two groups of participants were provided with an accurate external representation of the VE to use during navigation: one group was provided with verbal route directions and one group with schematic overhead maps (Appendix 1).

Although the present study is similar to that of Meneghetti and Pazzaglia (2021), it differs in several important ways. First, in this study participants were given the representations *during* navigation rather than beforehand. In terms of the verbal descriptions, the present study used route-based directional instructions whereas Meneghetti and Pazzaglia used a configural description of the environment with route information embedded within. There were also important differences between the two VEs. The VE used in the present study was a desktop VE, not a fully immersive VE, it required navigating 4 routes with many salient features like signs, trees, buildings, and cars, and the navigated routes included curved roads and turns of varying degrees. The two studies also used different measures for assessing spatial knowledge. In the present study, we used a within-route pointing task to assess elaborate route knowledge or local configuration knowledge, while Meneghetti and Pazzaglia used a route retracing task that simply required the participant to retrace the route they initially navigated. In terms of assessing global survey or configural knowledge, we used a complex between-route pointing task with many trials, while Meneghetti and Pazzaglia used only a single pointing judgment. Finally, we used a map completion task that required the participants to place all target buildings on a map in their relative locations whereas Meneghetti and Pazzaglia used a path drawing task that required participants to draw the single path they navigated.

Despite the differences between the present study and that of Meneghetti and Pazzaglia (2021), we predicted that we too would see a benefit for navigating with the external map representation compared to having no external representation. Specifically, we hypothesized that viewing the map during their second navigation experience in the VE would allow people to develop more accurate global survey or configural knowledge as they fitted what they saw sequentially to an overall conceptualization. Specifically, we predicted that after an initial baseline navigation experience, participants would demonstrate greater global configural knowledge gains (increased between-route pointing performance) during a second navigation experience if they had access to an external representation of the environment, especially a map or perhaps also verbal instructions, compared to having no external support tool.

## Method

### Participants

A sample of 139 participants enrolled in an undergraduate psychology course were recruited for the two-part study in exchange for class credit. Twenty-two participants were dropped because they did not return for the second session and two additional participants were dropped due to a technology failure during one of the sessions. The final sample consisted of 115 participants (*M*_*age*_ = 20.73 years, *SD* = 3.52 years; see Additional file 1: Table S4 for a breakdown of sex by condition).

### Pre-registered sample size justification and power analysis

As specified in our preregistration (https://osf.io/sfmpk), our sample size was based on data collected in a similar sample of undergraduates (Nazareth et al., 2019). In that study, a group of communication students completed Virtual Silcton at two timepoints (before and after a semester of mostly communications classes) and improved from Time 1 (*T*1) to Time 2 (*T*2) with a small effect size (*d* = 0.13), whereas a group of Geographic Information Systems students (before and after a semester of geosciences classes) improved with a medium effect size (*d* = 0.56). We interpreted these effect sizes as roughly upper and lower bounds of the effect sizes we should expect in any experiment on improving navigation performance on this task.

We used a sequential analysis plan (Lakens, 2014), in which batches of participants are collected, statistical tests calculated, and an a priori decision is made whether to recruit another batch of participants based on the results. A sequential analysis plan is ideal here because our range of possible effect sizes was large. We tested our intervention using two effect size thresholds. For the first threshold, we based our effect size calculation on the upper bound (*d* = 0.56). To obtain an effect size at least this large with 80% power, we estimated that we would need 46 participants per group (verbal/map/control). If, after testing 46 participants, the effect size of the intervention greater than control was *d* = 0.56 or higher, we would stop recruiting and publish the results.

However, the effect of one short intervention is likely to be much lower than a semester-long course. In essence, we want to know whether the intervention improves spatial knowledge more than test–retest alone (*d* = 0.13 from Nazareth et al., 2019). We calculated the smallest effect size we could obtain with a significantly greater effect than test–retest given our resource constraints, resulting in *d* = 0.42. If the effect size from the first batch was below* d* = 0.56 but above *d* = 0.42, we would collect an additional 46 participants.

A third possible outcome after the initial batch was an effect size less than *d* = 0.42. In this case, we would not recruit a second batch of participants, because we would be unlikely to obtain an effect significantly greater than test–retest even after additional data. Ultimately, after collecting 46 participants in each condition, the effect size obtained was less than *d* = 0.42 and thus we discontinued data collection with a sample of 139.

## Materials

### Virtual navigation paradigm and navigation measures

The experiment was administered on the same computer set-up and equipment as in Study 1. In the present study, participants completed the Virtual Silcton tasks at two timepoints (*T*1 and *T*2). Each timepoint consisted of two phases: a learning phase and a testing phase.

At T1, participants completed the learning phase (passively travel along each of the four routes via arrows indicating the direction that should be followed: main route A, main route B, connecting route C, connecting route D), then the testing phase (pointing and model-building tasks). The goal of this initial learning phase was to make sure all participants had equal exposure to the VE prior to using an external representation to guide their navigation to reduce the possible interference in visuospatial working memory caused by using a map. Then, based on their performance at *T*1, participants were assigned to one of three experimental conditions at *T*2: map representation, verbal representation, or control. To allow for a balanced distribution of good and bad navigators across conditions, assignment was based on which of three groups the participant’s pointing error scores fell into (integrators, non-integrators, or imprecise navigators; determined by a large existing sample of previously collected participants; Weisberg & Newcombe, 2016). Once coded into one of the three navigator groups, participants were pseudo-randomly assigned to the map representation group, verbal representation group, or control group at *T*2 to ensure the three navigator groups were equally represented in each condition (see Table 5). At *T*2, the learning phase was altered in a way determined by experimental condition. The testing phase was held constant.

**Table 5:** Total number of participants by navigator profile and condition in study 2

	Control	Verbal	Map	Total
Imprecise	13	10	10	53
Non-integrator	18	17	18	53
Integrator	9	10	10	29

#### Map representation condition

In the map representation condition, participants were given overhead view maps of the entire environment. For all four routes (two main and two connecting), there were no arrows in the VE to indicate the direction they should navigate. Rather, participants were given maps that were marked with the routes they were to follow and told to use these maps to navigate. Participants were given different maps for each route they completed (4 in total). Each map showed an overhead view of the entire environment, but only marked the directions (with a line) they needed to take for that specific route. As during the learning phase at *T*1, participants walked from the start of each route to the end and then back to the start. Each route was surrounded by invisible walls to keep the participant along the specified path. Participants had access to the map needed for each route, while they were navigating it (e.g., they had the map for route 1 while navigating route 1, etc.). The maps were printed in color on 8.5 × 11-inch pieces of paper that were clipped to a stand next to the computer monitor that participants were able to see while navigating without having to hold it or look down on the table in front of them. The experimenter changed the presented map between each route so that it matched the route they needed to navigate. See Appendix 1 for an example of one overhead map given to participants.

#### Verbal representation condition

In the verbal representation condition, participants were given step-by-step route instructions for how to navigate each route in the VE. As in the map representation condition, there were no arrows in the VE indicating the route, and instead, participants were told to read and follow the written directions to navigate each route. The verbal instructions were printed out on 8.5 × 11-inch pieces of paper that were clipped to a stand next to the monitor so the participant could read them while navigating. The experimenter changed the verbal instructions between routes to match the directions needed for navigating each route. See Appendix 1 for an example of a set of verbal directions given to participants.

#### Control condition

In the control condition, participants passively navigated through all four routes in the same manner as they did at *T*1. That is, each route was marked with red arrows they were to follow in the VE, and participants were not given any external representations to aid in their navigation.

### Psychometric and self-report measures

All participants completed a battery of six psychometric and self-report measures. Four of the measures were the same as those used in Study 1. These included the WRAT-4 as a verbal measure, running span and backwards digit span as measures of working memory capacity, and the OSIVQ as a spatial thinking self-report measure. Participants also completed a measure of spatial perspective taking, and a second spatial thinking self-report measure. All measures were administered at *T*1, except for the working memory measures which were administered at the end of *T*2.

#### Perspective-Taking Task—Adult (PTT-A)

The perspective task was a computerized version of a spatial perspective-taking task adapted from Frick et al. (2014). This task required participants to visualize what photographs would look like when taken from cameras placed at different positions and angles relative to their viewpoint. The original task was designed for children, and thus, the version used in the present study was adapted in difficulty level for use in adult populations (Brucato et al., 2023; Cronbach’s *α* = 0.79). In each item, participants were shown an image of a toy character near an arrangement of three objects. The participant’s task was to determine what that arrangement of objects would look like from the toy character’s perspective. For each item, there were 8 possible response options of which only a single option was correct. Participants first completed three practice items with correct answer feedback. After completing the practice items, participants were given three minutes to complete 16 items. Items always contained 3 objects in the layout and the angular difference between the toy characters and the participants’ perspective varied between 0°, 45°, 90°, 135°, and 180° to the right and or left. Each of the eight angular differences (0°, 180°, to the right 45°, 90° or 135°, and to the left 45°, 90° or 135°) was presented four times. Accuracy was recorded via participants’ click selection for each item and scores were calculated at the total number of items correctly answered (Cronbach’s *α* = 0.80 in the present sample).

#### Navigation Strategies Questionnaire

The Navigation Strategies Questionnaire (NSQ, Brunec et al., 2018) is a 14-item survey that is used to assess participants propensity for cognitive map-based navigational strategies. The items ask participants questions related to their use of maps while navigating, including their ease with using maps and their preferences toward maps or verbal instructions. Each response has an answer corresponding to a map-based navigation strategy or characteristic and one corresponding to a non-map or scene-based strategy. The mapping tendency was calculated as the difference between the number of map-based answers and non-map-based answers. Some questions have a third alternative, which was not coded. Higher scores on this measure indicate preferences toward scene-based strategies, and lower scores indicate preferences toward map-based strategies. Reliability for this measure was not reported in the original studies by Brunec and colleagues (Brunec et al. 2019, Brunec et al., 2018) and in the current sample Cronbach’s *α* = 0.50.

## Procedure

Participants were run individually in two sessions. At *T*1, participants signed a consent form informing them about the two-timepoint study. Participants could opt out at any point during the study. After consenting to participate, they completed a short demographic form and then began the VE navigation task. Participants were instructed to navigate the two main routes and two connecting routes in Virtual Silcton, and to complete the pointing and model-building tasks. Upon completing the virtual navigation paradigm, participants completed the OSIVQ and NSQ online via Qualtrics. Next the experimenter administered the PTT-A task on the computer via MATLAB, followed by the WRAT-4. At the end of *T*1, participants were scheduled for the second session approximately 10–14 days later. Session 1 took roughly 1 h to complete.

At *T*2, participants began the VE navigation paradigm and, as at *T*1, were instructed to navigate the two main routes and two connecting routes in Virtual Silcton, and to complete the pointing and model-building tasks. Participants in the control condition completed the VE navigation task in the same manner as they did at *T*1. Participants in the map representation condition were given a physical overhead map of the VE and were instructed to follow the indicated paths on the map. Participants in the verbal representation condition were given a set of written instructions for how to navigate through each route. In all three conditions, participants completed the pointing and model-building tasks during the test phase. These measures were identical to those administered at *T*1. Lastly, participants were given a brief 5-min break and then completed the two working memory measures on the computer. Session 2 took approximately 1.5 h to complete. The entire study from start to finish took approximately 2.5 h across both sessions and all participants were able to complete all given tasks in the allotted time.

## Results

To ensure there were no baseline differences across the three experimental conditions, we conducted one-way ANOVA tests with condition as the independent variable and our psychometric and self-report measures as dependent variables. These analyses revealed no baseline differences in working memory capacity (*F*(2, 103) = 1.13, *p* = 0.33, *ƞ*^2^ = 0.02, BF_10_ = 0.10), verbal fluency (*F*(2, 112) = 0.09, *p* = 0.91, ƞ^2^ = 0.002, BF_10_ = 0.03), navigation strategy preference (*F*(2, 112) = 0.36, *p* = 0.70, *ƞ*^2^ = 0.006, BF_10_ = 0.04), cognitive style (object: *F*(2, 112) = 0.15, *p* = 0.86, ƞ^2^ = 0.003, BF_10_ = 0.04; spatial: *F*(2, 112) = 0.26, *p* = 0.77, *ƞ*^2^ = 0.005, BF_10_ = 0.04; and verbal: *F*(2, 112) = 0.19, *p* = 0.83, *ƞ*^2^ = 0.003, BF_10_ = 0.04), or perspective taking (*F*(2, 109) = 1.93, *p* = 0.15, *ƞ*^2^ = 0.034, BF_10_ = 0.057) as a function of representation condition.

### Confirmatory analyses

Our principal and pre-registered hypothesis was that navigating with the external representations at *T*2 would promote greater spatial knowledge acquisition compared to the control condition. Specifically, we predicted that participants in the map and verbal conditions would outperform participants in the control condition on between-route pointing. To test our main hypotheses, as indicated by our preregistration (https://osf.io/sfmpk) we ran two separate two-tailed independent samples t tests. The first t test compared between-route pointing improvement (*T*1 to *T*2) in the map condition to the control condition. This analysis revealed no significant difference between the groups, *t*(76) = 0.23, *p* = 0.82, *d* = 0.05, BF_10_ = 0.24. The second t test compared between-route pointing improvement in the verbal representation condition and the control condition and again revealed no difference, *t*(75) = 0.35, *p* = 0.73, *d* = 0.08, BF_10_ = 0.25. Overall, these results did not support the hypotheses that providing learners with an external representation aid during navigation would support spatial integration and global configural knowledge development.

### Exploratory analyses

We conducted the following exploratory analyses to determine whether the representation conditions had any discernible effect on performance.^4^

#### Pointing task

We examined differences between participants based on their performance on the two types of pointing trials, between-route or within-route (see Table 6). We examined performance on the within-route trials by conducting a 2 (timepoint: *T*1, *T*2) X 3 (condition: map, verbal, control) repeated measures ANOVA. This analysis revealed a main effect for timepoint such that error on within trials was lower at *T*2 than *T*1, *F*(1, 112) = 29.46, *p* < 0.001, *η*_p_^2^ = 0.21. There was no main effect for condition (*F*(2, 112) = 0.17, *p* = 0.84, η_p_^2^ = 0.003), and there was no interaction between timepoint and condition,* F*(2, 112) = 0.93, *p* = 0.40, *η*_p_^2^ = 0.016. The same analysis was run for the between-route trials and again, there was a significant main effect of timepoint indicating less error at *T*2 than *T*1, *F*(1, 112) = 38.97, *p* < 0.001, *η*_p_^2^ = 0.26. There was no main effect of condition (*F*(2, 112) = 0.19, *p* = 0.82, *η*_p_^2^ = 0.003) and no interaction between condition and timepoint, *F*(2, 112) = 0.07, *p* = 0.93, *η*_p_^2^ = 0.001.

**Table 6:** Means and standard deviations for VE performance by condition and timepoint

	Control		Verbal		Map	
	Time 1 M (SD)	Time 2 M (SD)	Time 1 M (SD)	Time 2 M (SD)	Time 1 M (SD)	Time 2 M (SD)
Within-route error	33.04 (19.29)	24.83 (15.19)	32.00 (19.38)	27.44 (19.56)	30.40 (15.00)	24.74 (14.46)
Between-route error	61.54 (16.82)	50.99 (23.21)	58.35 (19.55)	49.19 (22.05)	59.84 (16.88)	50.23 (18.29)
Model-building accuracy	0.49 (.29)	0.57 (.30)	0.45 (.30)	0.55 (.31)	0.49 (.29)	0.77 (.21)

#### Model-building task

We examined whether model building improved as a function of representation condition (Table 5). Differences in model-building performance were modeled as a function of timepoint and condition, using a 2 (timepoint) X 3 (condition) repeated measures ANOVA. In addition to a main effect of timepoint such that participants generated more accurate models at *T*2 than at *T*1, *F*(1, 112) = 34.23, *p* < 0.001, *η*_p_^2^ = 0.23, and a marginal main effect of condition, *F*(2, 112) = 2.87, *p* = 0.06, *η*_p_^2^ = 0.05, we observed a significant interaction between timepoint and representation condition, *F*(2, 112) = 6.59, *p* < 0.002, *η*_p_^2^ = 0.11. Follow-up post hoc contrasts revealed that the map condition showed greater improvement from *T*1 to *T*2 (*M* = 0.29, *SD* = 0.26) than participants in either the verbal (*M* = 0.09, *SD* = 0.30; *t*(73) = 2.98, *p* = 0.004, *d* = 0.69, BF_10_ = 9.73) or control conditions (*M* = 0.08, *SD* = 0.28; *t*(76) = 3.42 *p* = 0.001, *d* = 0.78, BF_10_ = 30.12) and that there was no difference in *T*1 to *T*2 gain between the verbal and control conditions, *t*(75) = 0.24, *p* = 0.81, BF_10_ = 0.24. Providing an accurate map led participants to recreate a more accurate map of Virtual Silcton.

#### Navigator types

**Fig. 2:**
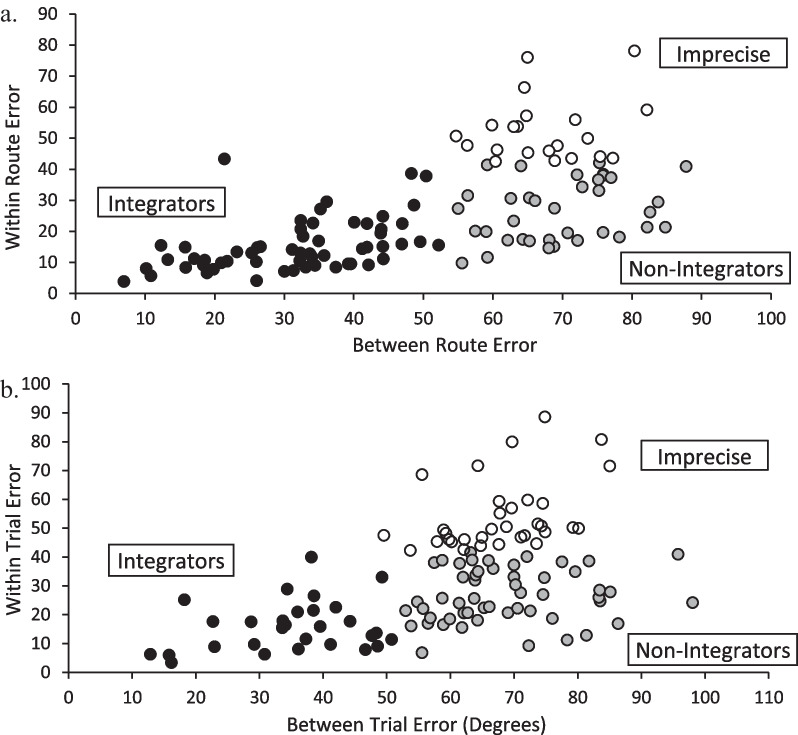
Navigator profiles at *T*1 and *T*2 for Study 2. *Note.* The top panel **a** shows navigator profiles at *T*1, and the bottom panel **b** shows navigator profiles at *T*2

As previously indicated, all participants were classified into one of three navigator types based on their scores on the within- and between-route pointing tasks. Following the method of Weisberg et al. (2014), we clustered participants based on their scores on between and within trials at both *T*1 and *T*2 using SPSS 28 statistical software’s two-step cluster analysis algorithm with log-likelihood as the distance measure. The two-step algorithm first assigns individual values into pre-clusters, which in turn are clustered together to maximize the log-likelihood of a case belonging to that cluster. We constrained the number of clusters to three to represent the navigator types found in prior research (integrator—good on within- and between-route pointing tasks; non-integrator—good on within- and bad on between-route pointing; imprecise—bad on within- and between-route pointing tasks). This grouping for *T*1 and *T*2 is displayed across two panels in Fig. 2.

Although there was no overall effect of representation condition, we examined the effect of condition across the navigator types to see whether navigators of different ability levels used the representations effectively. We conducted a 2 (pointing task: within change; between change) × 3 (representation condition: map, verbal, control) × 3 (navigation type: integrator, non-integrator, imprecise) repeated measures ANOVA. We did not observe a main effect of representation condition (*F*(2, 106) = 0.39, *p* = 0.68, η_p_^2^ = 0.01), nor significant two-way interactions between pointing task improvement and representation condition (*F*(2, 106) = 0.09, *p* = 0.92, *η*_p_^2^ = 0.02) or between representation condition and navigator type (*F*(4, 106) = 0.31, *p* = 0.87, *η*_p_^2^ = 0.01). There was no main effect of pointing task (*F*(1, 106) = 1.87, *p* = 0.18, η_p_^2^ = 0.02). Within- and between-route pointing error did not differentially improve. There was a significant main effect of navigator type (*F*(2, 106) = 3.88,* p* = 0.024, *η*_p_^2^ = 0.07) such that non-integrators showed greater *T*1 to *T*2 pointing task improvement than integrators (*p* = 0.018), but there was no difference in *T*1 to *T*2 improvement between integrators and imprecise navigators (*p* = 0.40) or between non-integrators and imprecise navigators (*p* = 0.34). There was, however, a significant two-way interaction between pointing task improvement and navigator type, *F*(2, 106) = 22.49, *p* < 0.001, *η*_p_^2^ = 0.30. The three-way interaction between pointing task improvement, navigator type, and representation condition did not approach significance, *F*(4, 106) = 1.70, *p* = 0.16, *η*_p_^2^ = 0.06.

**Fig. 3:**
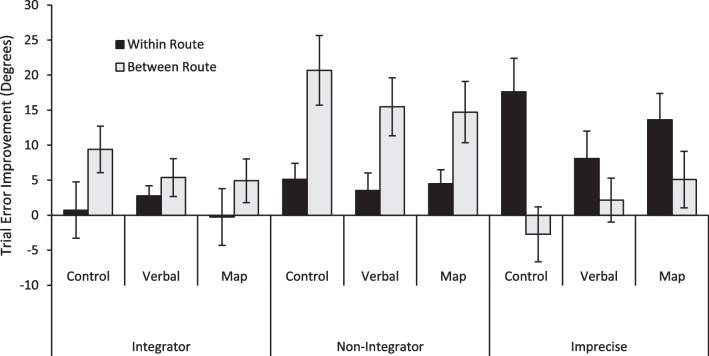
Within- and between-route pointing improvement as a function of navigator type in Study 2

To follow up the two-way interaction between pointing task improvement and navigator type, two one-way ANOVAs were conducted (see Fig. 3) 5. First, a one-way ANOVA examining within-route pointing improvement from *T*1 to *T*2 as a function of navigator type was significant, *F*(2, 112) = 10.75, *p* < 0.001, *ƞ*^2^ = 0.16. Follow-up pairwise comparisons with Bonferroni correction indicated no difference in within-route improvement between integrators and non-integrators (*p* = 0.60), but imprecise navigators showed greater within-route improvement than non-integrators (*p* = 0.001, *f* = 1.14) and integrators (*p* < 0.001, *f* = 1.36). A one-way ANOVA examining between-route pointing improvement from *T*1 to *T*2 as a function of navigator type was also significant, *F*(2, 112) = 11.95, *p* < 0.001, ƞ^2^ = 0.18. Follow-up pairwise comparisons with Bonferroni correction indicated no difference in between-route improvement between integrators and imprecise navigators (*p* = 0.51), but non-integrators showed greater between-route improvement than imprecise navigators (*p* < 0.001, *f* = 1.81) and non-integrators showed greater improvement than integrators (*p* = 0.01, *f* = 1.15).

## Discussion

Our primary question for this study was whether providing an external representation in the form of allocentric maps or route-based verbal directions during navigation would result in improved configuration knowledge of a novel VE. Counter to our pre-registered hypothesis, we did not observe greater improvement in participants’ global spatial configuration knowledge acquisition, as measured by the between-route pointing task, when they had access to either a verbal or map-based representation of Virtual Silcton as they traveled along the routes for a second time. However, we did find that participants who received a map could accurately recreate the layout of landmarks in a model-building task; but those same participants could not perform more accurately on a pointing task. This finding is somewhat surprising given that prior research has found benefits for navigating with map-based representations (Gardony et al., 2013; Ishikawa et al., 2008; Münzer et al., 2012, 2020) and with verbal route-based representations (Hund & Minarik, 2006; Saucier et al., 2002; Streeter et al., 1985).

Beyond our pre-registered hypothesis, we did not observe greater improvement for those with an aid compared to the control group on any navigation task by any group of navigators. However, across all groups including the control group, we found robust improvement in within-route pointing, between-route pointing, and model-building performance from *T*1 to *T*2. This finding aligns with previous evidence suggesting that mental representations of space become more refined with greater exposure to the environment (Golledge & Spector, 1978; Muffato et al., 2021; Muffato & Meneghetti, 2020; Thorndyke & Hays-Roth, 1982).

Further, our exploratory analyses suggested that while all groups saw spatial knowledge improvements from *T*1 to *T*2, navigators of differing ability levels improved on distinct aspects of spatial knowledge. In particular, we saw that the least skilled navigators, or those we called imprecise navigators, improved significantly on the within-route pointing trials, while the moderately skilled navigators, or those we called non-integrators, improved on the between-route trials. Although exploratory in nature, these results are interesting because they suggest that repeated exposure to an environment may help less skilled navigators develop improved route knowledge or local configuration knowledge, but not more global survey or configuration knowledge. On the other hand, moderately skilled navigators may already have relatively accurate route or local configuration knowledge, and thus the increased exposure to the environment supports improvements in their global survey knowledge. Additionally, these findings seem somewhat aligned with the idea that one must develop spatial knowledge sequentially, beginning with landmark knowledge, then route knowledge, and then finally survey knowledge (Siegel & White, 1975). However, the present data cannot speak directly to this idea and instead may indicate that previously reported individual differences in spatial learning (Weisberg et al., 2014) should be characterized as differences in learning curves—poorer navigators do learn spatial environments, but at slower overall rates and with different spatial knowledge crystallizing in different stages. This interpretation aligns with Montello’s (1998) framework of the structure and developmental course of spatial knowledge.

## General discussion

Navigational aids such as maps and verbal instructions are important tools for conveying spatial information and guiding people through space. Maps can be especially useful for facilitating configural understanding of space, compared to direct experience, because they provide information about an entire layout simultaneously and the relative locations of important landmarks (Ishikawa et al., 2008; Thorndyke & Roth, 1982). Verbal instructions, on the other hand, may be especially useful for supporting navigation because they can support the formation of mental representations with spatial properties (Brunyé et al., 2007; Gyselinck & Meneghetti, 2011), but are less likely to interfere with spatial working memory because they involve a multimedia learning approach where the same information is provided using visual and verbal formats as opposed to a single format (Mayer, 2019).

Across two studies, we examined the impact of map-based representations and verbal route-based representations on participants development of spatial knowledge of a complex spatial VE. In Study 1, the main question was whether *generating* one’s own externalization could improve the quality of one’s internal representation of the spatial environment. Overall, we found that generating one’s own external representation, whether visual or verbal, did not improve within- or between-route pointing or model building. However, we did see that pointing and model-building performance was related to the quality of the external representation participants generated. That is, participants who generated higher quality maps that attempted to represent both main routes and the connecting route, also demonstrated better pointing and model-building performance. This result suggests that generating an external representation may serve better as a measure of one’s cognitive map as opposed to a tool for supporting cognitive map development. However, another interpretation is that these representations do not actually serve as tools for supporting spatial integration or the development of configural knowledge, but rather serve as another measure of one’s internal cognitive map.

One explanation for why we did not see a benefit for generating external representations may be due to participants not getting feedback. In studies on generating representations from science text, benefits from drawing are usually seen when participants get feedback and are allowed to integrate that feedback into updating their representation (Fan, 2015; Gagnier et al., 2017; Van Meter & Garner, 2005). In our study, participants were asked to create a representation, but were never explicitly shown a correct map or description and thus, were never able to update their own mental map with this corrective feedback. Participants in this study were able to freely explore the environment after generating their external representation. The goal of this free exploration period was for participants to be able to explore parts of the environment they felt they needed more experience with to better understand the whole VE. However, due to a technology failure, the log files during this phase were lost and we were unable to examine the free exploration data. Future research should directly examine the impact of map drawing activities on cognitive map development in situations where participants are given feedback on their maps and allowed to revise them. An interesting direction for future research would be to have participants go through multiple rounds of navigation, representation generation, feedback and updating. Perhaps multiple cycles of navigating, then generating a representation, then navigating again, and then updating that representation through multiple iterations would allow participants to form an internal cognitive map that includes both allocentric and egocentric perspectives.

Navigating almost always requires that multiple pieces of spatial information be maintained and integrated and previous work has demonstrated a relationship between working memory and the acquisition of spatial knowledge through navigation (Blacker et al., 2017; Weisberg & Newcombe, 2016; Wen et al., 2011). Further, previous work has demonstrated that individual differences in working memory performance are more evident when load is high (Cusack et al., 2009; Linke et al., 2011). In the present study, participants were expected to navigate the new and novel environment while also creating an integrated spatial mental representation. For individuals with a poor sense of direction or less efficient concurrent-processing capacity (i.e., working memory capacity), this dual requirement may have created a bottleneck in processing that impacted their ability to generate an integrated representation or benefit from the act of generating an external representation. Thus, like the design in Study 2, future research should also consider providing participants with an initial baseline guided navigation experience to familiarize them with the VE prior to completing a target navigation experience during which they complete externalization activities to lessen the impact of cognitive constraints in processing capacity.

In Study 2, the main question was whether participants need to be provided with an accurate representation of the spatial environment while navigating in order to improve their spatial knowledge about the VE. A key finding from Study 2 indicated that participants who received a map were able to reconstruct the map of the environment, but this spatial knowledge did not translate to being able to take make more accurate within-route or between-route pointing estimation within the environment. That is, participants who were given maps to aid their navigation showed improved model-building performance compared to participants given verbal instructions or no external aid but were no better at the within-route or between-route pointing task. This suggests that using a well-suited and valid externalization will not necessarily transfer across perspectives or less-related tasks. There are several possible explanations for the current findings and the discrepancies between them and other experiments.

Some studies have found benefits for maps or verbal instructions in VE navigation scenarios, but these studies have differed from the present study in many ways. For example, Meneghetti and Pazzaglia (2021) obtained results showing that performance on a pointing task did not differ between those provided a map and those provided verbal instructions, but participants in the map group were better able to draw the path they navigated. In that study, participants were given the external representation before navigation, while in the present study participants were given the representations *during* navigation. This difference could have introduced visuospatial working memory interference and decreased participants spatial knowledge acquisition. In terms of the verbal descriptions, the present study used route-based directional instructions, whereas Meneghetti and Pazzaglia used a configural description of the environment with route information embedded within. This difference is important because in our study, we may have reinforced a route-based or egocentric perspective that was more difficult to transform into an allocentric perspective, which is important for being able to make accurate pointing estimations and complete global configural representations of target landmarks. There were also important differences between the two VEs such that the VE used in the present study was a desktop VE (not a fully immersive) that required navigating 4 routes with many salient features, and the navigated routes included curved roads and turns of varying degrees. In the study by Meneghetti and Pazzaglia, a more immersive experience with less perceptual features and less complexity may have made it easier for participants to integrate the egocentric navigation experience into an allocentric representation that could be used to guide their configural knowledge. Finally, the present study and the study by Meneghetti and Pazzaglia (2021) used different measures for assessing spatial knowledge. In the present study, we used a within-route pointing task to assess more elaborate route knowledge or local configuration knowledge, while Meneghetti and Pazzaglia used a route retracing task that simply required the participant to retrace the route they initially navigated. In terms of assessing survey or global configural knowledge, we used a complex between-route pointing task with many trials, while Meneghetti and Pazzaglia used only a single pointing judgment. Finally, we used a map completion task that required the participants to place all target buildings on a map in their relative locations, whereas Meneghetti and Pazzaglia used a path drawing task that required participants to draw the single path they navigated.

Similarly, in a recent study by Münzer et al. (2020), participants navigated through a VE presented across three screens simultaneously, but only navigated a single route with 5 target buildings, in contrast to our study that used 4 routes with a total of 8 target buildings divided across the two main routes. Because they only used a single route, the pointing task used by Münzer et al (2020) aligns most closely with the within-route trials in our study and they had no measure of global integration, or between-route pointing judgments. Further, the model-building task they used provided a map with roads that participants placed target buildings along. In our study, the model-building task only provided a blank space with no roads or other environment information. An important direction for future research is to explore these differences in navigation experiences more systemically. For example, it is possible that maps or verbal instructions are useful for supporting cognitive map development only up to a certain point. That is, once an environment includes more than one or two routes and requires integration of these multiple routes, do maps or verbal descriptions become more or less useful?

One interpretation of the findings in this study is that knowledge about the directions between individual landmarks is not easily extracted from maps, or verbal directions. We call this the *transformation hypothesis* because the difficulty lies in transforming the information on the map or verbal instructions into a single integrated mental representation that includes both allocentric and egocentric information. Despite having an accurate map to scaffold their spatial knowledge acquisition, participants did not perform any better on a task measuring their ability to make configural pointing estimations (local or global) than simply a second time traveling the routes.

One other potential explanation for the general lack of spatial knowledge acquisition differences between representation conditions is that our intervention was not sufficient to overcome additional exposure to the environment itself. In an exploratory analysis from Study 2, we observed differences in improvement over time depending on navigation ability and pointing trial type regardless of the additional representation they received. The improvement was also specific to navigation ability. Integrators, who perform well overall on the Virtual Silcton, showed minor improvements on the between-route pointing trials (their within-route pointing trials are already nearly at ceiling). Non-integrators and imprecise navigators showed opposite patterns of improvement. Non-integrators improved from *T*1 to *T*2 on the between-route pointing (again, their within-route pointing trials are also near ceiling). Imprecise navigators showed little improvement on between-route pointing trials, but their performance improved on within-route pointing trials. Although these results should be interpreted with caution due to the low sample size of each navigator profile across the three conditions, this longitudinal finding adds to the mounting evidence that within- and between-route pointing trials require distinct knowledge, improve at different rates, and may have different cognitive underpinnings (Hilton et al., 2021; Montello, 1998; Siegel & White, 1975). These gains, though not specific to the interventions tested here, support a model of spatial learning in which people learn environments at different rates. Additional experience with the environment itself may have been just as useful as external representations. Future research should take into consideration individual differences in other cognitive capacities that have been shown to relate to successful navigation including perspective taking and spatial orientation skill.

Overall, a limited number of studies have examined the effects of instruction or training for improving cognitive mapping and in general the results have not been overwhelmingly positive. For example, in a study by Wen, Ishikawa and Sato (2014), participants were given instructions regarding how to learn about routes (either verbal or spatial). Their results demonstrated no effect of instruction type on route learning for either individuals with a poor sense of direction or participants with a good sense of direction. In terms of survey learning, they found that both types of instruction during navigation actually harmed survey learning for participants with a good sense of direction. Similarly, in a more recent study by Ishikawa and Zhou (2020), training in allocentric spatial updating also did not result in substantial improvements in cognitive mapping for individuals with poor sense of direction. Specifically, the training did not improve the accuracy of distance judgments or sketch map accuracy. There was an improvement in direction estimates, although the effect was small and participants with poor sense of direction still performed lower than participants with average sense of direction.

Taken together, results from prior research and the results of the two present studies speak to the challenge of using external representations to support improved navigation behavior, improved cognitive map development, and the strength of individual differences in spatial navigation behavior. Future research should continue to examine how, when, and for whom external representations can be used to support navigation.

### Supplementary Information

**Additional file 1.** Supplementary tables and analyses.

## Data Availability

The datasets used and/or analyzed during the current study are available from the corresponding author on reasonable request.

## Appendix 1

### Route A: verbal instructions

- On your left you will see BATTY HOUSE, to your right you will see a grove of trees.
- Follow the path straight ahead of you toward a gas station called LYNCH STATION.
- On your right you will see a handicap parking area, continue past this area and take the right at the corner.
- As you come around the corner, you will be on a road between two large buildings. As you continue walking, you will see that the building to your right is HARRIS HALL, and the one to your left is the Art Building.
- You will pass a hexagon-shaped building on your left and then Le Prince cafe on your right. As you continue along the path you will see City Hall on your left.
- Turn right and continue along the path. You’ll see the agriculture buildings on the right and athletic building on the left.
- As you pass the barn you will see a willow tree positioned at a fork in the road, and you will take a right.
- Once you’ve turned right, you will see HARVEY HOUSE to your left and Dr. Lobo’s office to your right. The finish line is directly ahead.

## Abbreviations

VE: Virtual environment
*T*1: Time 1
*T*2: Time 2
WRAT-4: Wide range achievement test
WAIS-III: Wechsler adult intelligence scale
WISC-IV: Wechsler intelligence scale for children
WMC: Working memory capacity
PTT-A: Perspective-taking task for adults
OSIVQ: Object-spatial imagery and verbal questionnaire
NSQ: Navigation Strategies Questionnaire
ANOVA: Analysis of variance
Tukey HSD: Tukey honestly significant difference test
GMDA: Gardony map drawing analyzer
SOT: Spatial orientation task
SBSOD: Santa Barbara Sense of Direction

## Footnotes

1 Although the sample contained a greater proportion of females overall, approximately one third of the sample in each condition was male. See Additional file 1 for a breakdown of participant sex by condition (Table S1).

2 Due to a glitch in the Silcton program, behavior during the free exploration phase was not logged and therefore no analyses were able to be conducted on this data.

3 Performance on the within-pointing, between-pointing, and model-building tasks was all highly correlated, as with previous studies using Virtual Silcton (between and within-pointing error *r* = .63, *p* < .001; within-pointing error and model building *r* = -.42, *p* < .001; and between-pointing error and model building *r* = -.59, *p* < .001). A table of correlations between VE performance measures and psychometric measures can be found in Additional file 1: (Table S3).

4 See Additional file 1: Table S5 for an exploratory correlational analysis.

5 As we were most interested in cross-condition analyses for this experiment and did not observe any group by condition interactions, navigator type analysis beyond what is reported in the manuscript is available in the Additional file 1.

## Open practices statement

The datasets used and analyzed during the current set of studies are available from the corresponding author on reasonable request. Study 2 was pre-registered on OSF (https://osf.io/sfmpk) where the design, hypotheses, and critical analyses of interest were described. Study 1 was not pre-registered; however, the authors are willing to make data and materials available upon request.
