# Supplementary material for: Using a picture (or a thousand words) for supporting spatial knowledge of a complex virtual environment
Source: Cogn Res Princ Implic. 2023 Jul 25;8:48. doi: 10.1186/s41235-023-00503-z (PMC10368603; doi:10.1186/s41235-023-00503-z)
Supplement: Supplementary file 1 — Additional file 1. Supplementary tables and analyses. [file 41235_2023_503_MOESM1_ESM.docx]

**Table S1**

*Total Number of Participants by Gender and Condition in Study 1*

|  | Sketch | Verbal | Control | Total |
| --- | --- | --- | --- | --- |
| Male | 17 | 17 | 25 | 59 |
| Female | 35 | 35 | 43 | 113 |
| Total | 52 | 52 | 68 | 172 |

**Table S2**

*Descriptive Statistics for Psychometric and Navigation Tasks by Condition in Study 1*

|  | Sketch | | Verbal | | Control | |
| --- | --- | --- | --- | --- | --- | --- |
|  | *M* | *SD* | *M* | *SD* | *M* | *SD* |
| WMC | .02 | 1.02 | .01 | .78 | .04 | .82 |
| SOT | 49.01 | 27.71 | 53.45 | 30.09 | 40.23 | 29.45 |
| SBSOD | 58.35 | 10.12 | 57.87 | 12.78 | 54.63 | 13.25 |
| WRAT | 46.13 | 5.00 | 45.75 | 6.23 | 46.64 | 4.95 |
| OSIVQ – O | 3.13 | .51 | 3.26 | .60 | 3.08 | .51 |
| OSIVQ – S | 2.64 | .58 | 2.60 | .59 | 2.71 | .63 |
| OSIVQ – V | 2.97 | .46 | 3.02 | .43 | 2.95 | .44 |

**Table S3**

*Correlations Among VE Performance and Psychometric Measures in Study 1.*

|  | 1. | 2. | 3. | 4. | 5. | 6. | 7. | 8. | 9. |
| --- | --- | --- | --- | --- | --- | --- | --- | --- | --- |
| 1. Between-route | --- |  |  |  |  |  |  |  |  |
| 2. Within-route | .63** | --- |  |  |  |  |  |  |  |
| 3. Model-build | -.59** | -.42** | --- |  |  |  |  |  |  |
| 4. WMC | -.33** | -.29** | .30** | --- |  |  |  |  |  |
| 5. SOT | .39** | .48** | -.34** | -.44** | --- |  |  |  |  |
| 6. SBSOD | -.28** | -.20** | .22** | .13 | -.01 | --- |  |  |  |
| 7. WRAT | -.29** | -.31** | .19* | .43** | -.27** | -.01 | --- |  |  |
| 8. OSIVQ–Spatial | -.33** | -.37** | .28** | .17^†^ | -.33** | .36** | -.01 | --- |  |
| 9. OSIVQ–Verbal | .01 | .05 | -.02 | .01 | .14 | .09 | .10 | -.04 | --- |
| 10. OSIVQ–Object | -.10 | -.14 | .14 | .08 | .01 | .37** | -.11 | .12 | .07 |

*Note.* ***p* < .01, **p* < .05, ^†^*p* < .10; WMC – working memory capacity composite score; SOT – Spatial Orientation Test; SBSOD – Santa Barbara Sense of Direction Scale; WRAT – Wide Range Achievement Test, Word Reading Subtest; OSIVQ – Object-Spatial Imagery and Verbal Questionnaire.

**Table S4**

*Total Number of Participants by Gender and Condition in Study 2*

|  | Map | Verbal | Control | Total |
| --- | --- | --- | --- | --- |
| Male | 10 | 10 | 9 | 29 |
| Female | 28 | 27 | 30 | 85 |
| Other | 0 | 0 | 1 | 1 |
| Total | 38 | 37 | 40 | 115 |

**Correlational analyses.** In addition to examining the impact of representation condition on spatial integration, we tested whether within-route pointing, between-route pointing, and/or model-building at Time 1 were predicted by our individual difference and cognitive style variables. As shown in Table S4, between and within-route pointing error and model-building were all highly correlated. These correlations align with those seen in previous studies using Virtual Silcton (e.g., Weisberg & Newcombe, 2016). In terms of participants’ self-reported navigation strategy preference, there was a moderate negative correlation between NSQ score and within and between-route pointing error such that more accurate participants were more likely to report using map-based strategies. On the other hand, there was a moderate positive correlation between NSQ and model-building which suggests that participants who generated more accurate models were more likely to report using scene-based strategies. There were no significant correlations between the Object and Spatial subscales of the OSIVQ and any of the navigation assessments. However, there were correlations between the Verbal OSIVQ subscale and within-route pointing error and the model-building task both indicating that more accurate participants scored higher on the Verbal subscale. We also examined correlations between the VE performance measures and performance on the PTTA perspective taking measure. More accurate perspective-taking was correlated with more accurate performance on the Virtual Silcton pointing tasks, but not model-building.

**Table S5**

*Correlations Among VE Performance and Psychometric Measures at Time 1 in Study 2.*

|  | 1. | 2. | 3. | 4. | 5. | 6. | 7. | 8. | 9. |
| --- | --- | --- | --- | --- | --- | --- | --- | --- | --- |
| 1. Between-route | --- |  |  |  |  |  |  |  |  |
| 2. Within-route | .49** | --- |  |  |  |  |  |  |  |
| 3. Model-build | -.62** | -.48** | --- |  |  |  |  |  |  |
| 4. NSQ score | -.34** | -.28** | .21* | --- |  |  |  |  |  |
| 5. OSIVQ–Spatial | .16^†^ | .11 | .01 | -.44** | --- |  |  |  |  |
| 6. OSIVQ–Verbal | -.12 | -.30** | .21* | .08 | .02 | --- |  |  |  |
| 7. OSIVQ–Object | .07 | .09 | -.03 | -.04 | .02 | -.23* | --- |  |  |
| 8. PTTA | -.26** | -.24* | .14 | .17^†^ | -.28** | .12 | .06 | --- |  |
| 9. WRAT | -.08 | -.26** | .07 | .01 | .06 | -.01 | .09 | .09 | --- |
| 10. WMC | -.33** | -.42** | .17^†^ | .04 | .01 | .07 | .19^†^ | .32** | .28** |

*Note.* ***p* < .01, **p* < .05, ^†^*p* < .10; NSQ – Navigation Strategy Questionnaire; OSIVQ – Object-Spatial Imagery and Verbal Questionnaire; PTTA – Perspective Taking Task Adult version; WRAT – Wide Range Achievement Test, Word Reading Subtest; WMC – working memory capacity composite score.

**Navigator Type Classification Change Analyses.** To explore changes in participants navigator type classification from T1 to T2, we conducted a chi-square test examining the frequency of category change (moved down a category, stayed in the same category, moved up a category) as a function of what category the participant was in at T1 (non-integrator or imprecise). Participants who were categorized as Integrators at T1 were excluded from this analysis because they all stayed in the same category. Overall, this resulted in a non-significant chi square test, *X*^2^(2) = 2.72, *p* = .26. As shown in Table S6 below, about 50% of the participants who were categorized as Non-integrators at T1 moved up to Integrators at T2, and about 50% of the participants who were categorized as Imprecise navigators at T1 moved up to Non-integrators at T2.

**Table S6**

*Frequency of Navigator Type Change from T1 to T2*

|  | Moved Down at T2 | Same at T2 | Moved up at T2 | Total N |
| --- | --- | --- | --- | --- |
| Non-Integrator at T1 | 4 (7.54%) | 22 (41.51%) | 27 (50.94%) | 53 |
| Imprecise at T2 | 0 (0.00%) | 16 (48.48%) | 17 (51.51%) | 33 |
| Total N | 4 | 38 | 44 | 86 |

**Pointing Task Improvement as a Function of Navigator Type Change.** For this analysis, we only included the 44 participants whose navigator type improved from T1 to T2. Of these 44 participants, 31 were categorized as Integrators at T2, and 13 were categorized as Non-Integrators at T2. We did not examine the performance of Imprecise navigators because they did not improve from T1 to T2.

To see if the participants categorized as Integrators at T2 improved enough to equate to the performance of Integrators at T1, a series of one-sample t-tests were conducted. These t-tests compared mean within and between pointing performance and model building performance at T2 to the average levels seen for Integrators at T1. There was no difference in mean within-pointing error between those who became Integrators at T2 (*M* = 17.35, *SD* = 7.25) and those who were Integrators at T1 (*M* = 15.96, *SD* = 8.75), *t*(30) = 1.07, *p* = .29, *d* = .19. There was no difference in mean between-pointing error between those who became Integrators at T2 (*M* = 36.52, *SD* = 11.11) and those who were Integrators at T1 (*M* = 35.02, *SD* = 10.75), *t*(30) = .751, *p* = .46, *d* = .14. There was also no difference in mean model building performance between those who became Integrators at T2 (*M* = .80, *SD* = .19) and those who were Integrators at T1 (*M* = .75, *SD* = .23), *t*(30) = 1.71, *p* = .10, *d* = .31. Together, these results indicate that participants who improved their spatial knowledge from T1 to T2 enough to be re-categorized as Integrators at T2 performed at a similar level to those who were already Integrators at T1.

To see if the participants categorized as Non-Integrators at T2 improved enough to equate to the performance of Non-Integrators at T1, another series of one-sample t-tests were conducted. Again, these t-tests compared mean within and between pointing performance and model building performance at T2 to the average levels seen for Non-Integrators at T1. There was no difference in mean within-pointing error between those who became Integrators at T2 (*M* = 30.34, *SD* = 10.31) and those who were Integrators at T1 (*M* = 26.57, *SD* = 9.12), *t*(12) = 1.32, *p* = .21, *d* = .36. There was no 71.84, *SD* = 7.23) and those who were N0n-Integrators at T1 (*M* = 68.67, *SD* = 10.65), *t*(12) = 1.58, *p* = .14, *d* = .44. There was also no difference in mean model building performance between those who became Non-Integrators at T2 (*M* = .49, *SD* = .21) and those who were Integrators at T1 (*M* = .44, *SD* = .23), *t*(12) = .83, *p* = .42, *d* = .23. Together, these results indicate that participants who improved their spatial knowledge from T1 to T2 enough to be re-categorized as Non-Integrators at T2 performed at a similar level to those who were already Non-Integrators at T1.
